# “Peanut saves the day”: an innovative solution to massive, cryptogenic haemoptysis—a case study

**DOI:** 10.1002/rcr2.754

**Published:** 2021-05-07

**Authors:** Sanjana Chetana Shanmukhappa, Srivatsa Lokeshwaran, Sunil Kumar K, Prakash Doraiswamy

**Affiliations:** ^1^ Department of Pulmonology Aster CMI Hospital Bengaluru India; ^2^ Department of Anaesthesia and Critical Care Aster CMI Hospital Bengaluru India

**Keywords:** Cryptogenic, haemoptysis, innovative, life‐threatening, massive

## Abstract

Massive (or life‐threatening) haemoptysis is a time‐sensitive emergency encountered by a physician that requires an interdisciplinary, collaborative effort to arrest the bleeding in a prompt and timely manner. Placement of an endobronchial Watanabe spigot (EWS) to halt haemoptysis is a relatively recent technique finding its wide application in airway pathology, with the current extension of its use to bronchial bleeding. However, the lack of immediate access to EWS gives rise to the need to innovate with day‐to‐day materials used in routine surgical practice and available in resource‐limited settings, which may serve the purpose of a spigot. In this report, we bring to light a case of life‐threatening, cryptogenic haemoptysis that was managed by a novel technique of using peanut gauze as a spigot resulting in a successful endobronchial tamponade.

## Graphical abstract

Massive (or life‐threatening) haemoptysis is a time‐sensitive emergency encountered by a physician. The lack of immediate access to an endobronchial Watanabe spigot gives rise to the need to innovate with day‐to‐day materials used in routine surgical practice and available in resource‐limited settings, which may serve the purpose of a spigot. In this report, we bring to light a case of life‐threatening cryptogenic haemoptysis that is managed by a novel technique of using peanut gauze as a spigot resulting in a successful endobronchial tamponade.

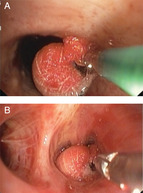

## Introduction

Haemoptysis or expectoration of blood originating from the lower respiratory tract can present as a benign, self‐limiting condition or a life‐threatening occurrence that requires prompt intervention. Massive haemoptysis is a time‐sensitive challenge for the physician to evaluate and manage in a step‐wise yet urgent manner. Without appropriate treatment, severe haemoptysis can lead to mortality in as many as 80% of the cases [1]. The cause of haemoptysis is wide‐ranging from airway/parenchymal‐related aetiology such as tuberculosis and neoplasm to vascular causes such as pulmonary embolism. However, about 30–40% of the cases remain without an identifiable aetiology and are labelled “cryptogenic” or “idiopathic” haemoptysis [2]. The sequence and timing of diagnostic procedures are determined by the patient's condition, whereas the treatment modalities are dependent on the availability of equipment and expertise. Placement of an endobronchial spigot to arrest haemoptysis is a relatively recent technique, finding its wide application in airway pathology and has been shown to be effective in as many as 78% of the cases of haemoptysis [3]. The challenge, however, lies in the immediate access to an endobronchial Watanabe spigot (EWS) for the procedure thereby giving rise to the need to innovate with day‐to‐day materials used in routine surgical practice which may serve the purpose of a spigot. In this report, we bring to light a case of life‐threatening cryptogenic haemoptysis that was managed by a novel technique of using peanut gauze as a spigot to arrest bronchial bleeding.

## Case Report

A 51‐year‐old male patient with a past medical history of type 2 diabetes mellitus on oral hypoglycaemic agents presented to the emergency room with complaints of cough with haemoptysis for the last five days following a recent trip to the state of Bihar, India. Haemoptysis was initially scanty in amount (about 25 mL/day) and increased to 200 mL/day for two days. There was no history of fever, shortness of breath, chest pain, skin rashes, jaundice, the passage of blood in stool or urine, loss of appetite, or loss of weight.

The patient had a history of exposure to tuberculosis at his workplace (the worker was sputum positive) one year ago. The patient also had a history of vertigo one year ago for which he received treatment. The patient had no history of hypertension, bronchial asthma, or ischaemic heart disease. He has no history of smoking or alcohol use. The patient had no significant family history.

On examination, the patient was afebrile and haemodynamically stable. Chest examination revealed left‐sided fine inspiratory crepitations. The rest of the local and systemic examinations were found to be within normal limits. The patient was admitted for further evaluation.

**Figure 1:**
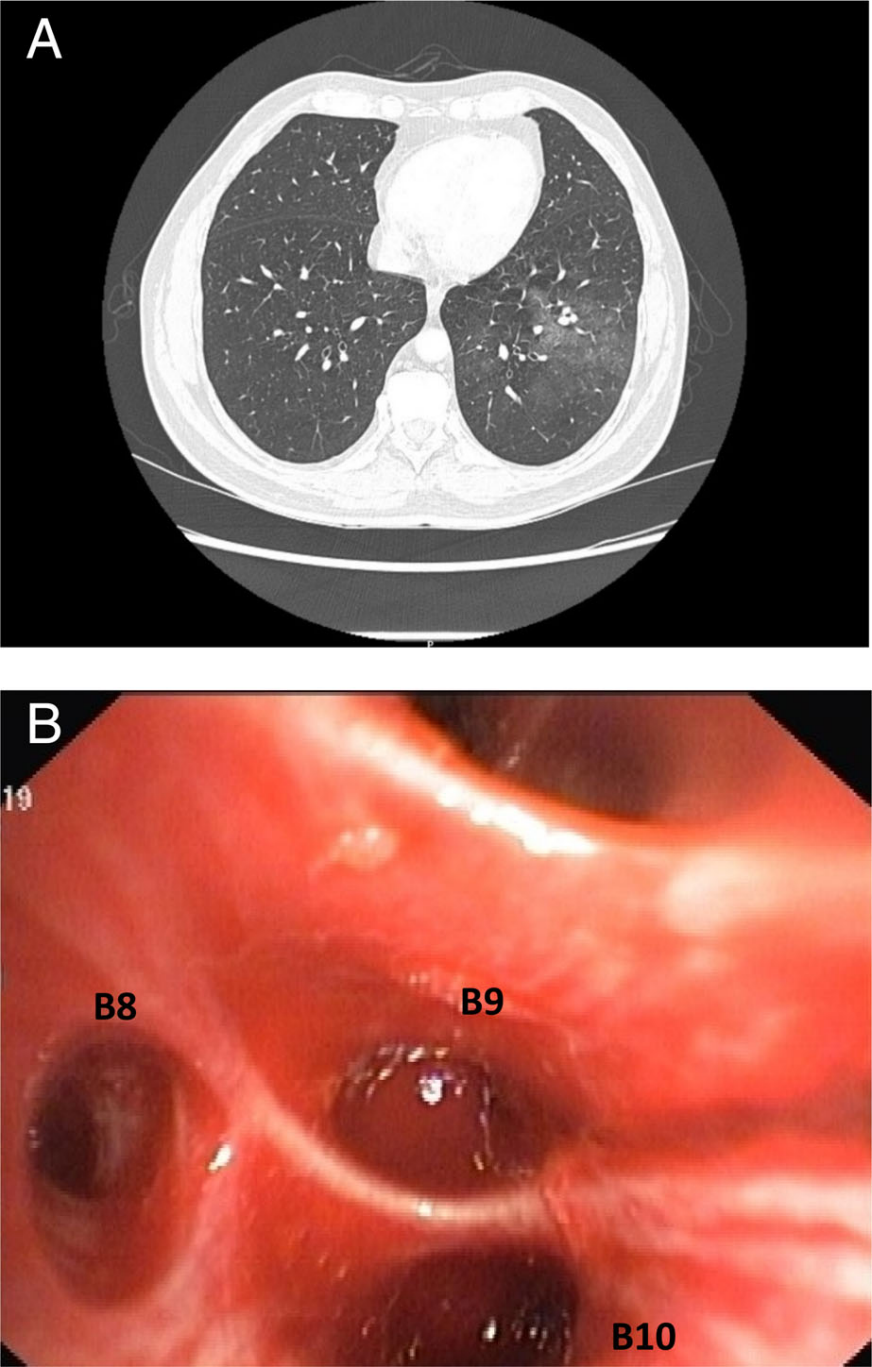
(A) Computed tomography (CT) scan showing ground‐glass opacity in the left lateral basal segment indicating the site of bleeding. (B) Bronchoscopy image showing pools of blood in the B9/B10 segments of the left lower lobe.

Laboratory investigations (coagulation profile, complete blood count, and renal and liver function tests) were found to be within normal limits. Computed tomography (CT) thorax with contrast showed ground‐glass densities in the right upper lung and left lower lung, features highly suggestive of alveolar haemorrhage (Fig. 1A). It also showed normal calibre bronchial artery at D5–6 level. This necessitated an interventional radiologist consultation to assess the feasibility of bronchial arterial embolization (BAE) to arrest the bleeding. In this case, the interventional radiologist ruled out any hypertrophied bronchial arteries or collaterals. This precluded the possibility of BAE. Echocardiography was normal with no evidence of pulmonary embolism, clots, or vegetations. The patient was initiated on intravenous tranexamic acid, cough suppressants, and empirical broad‐spectrum antibiotics, and was observed closely.

**Figure 2:**
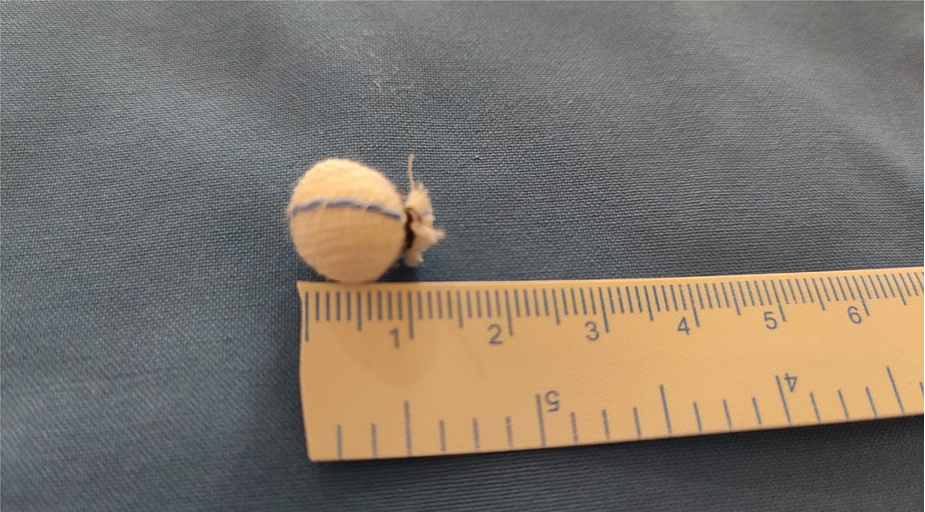
The spigot substitute: a gauze ball made up of gauze pads is rolled into the shape of a peanut and secured with a regular silk thread tied around its neck. Approximate size of 1 cm.

**Figure 3:**
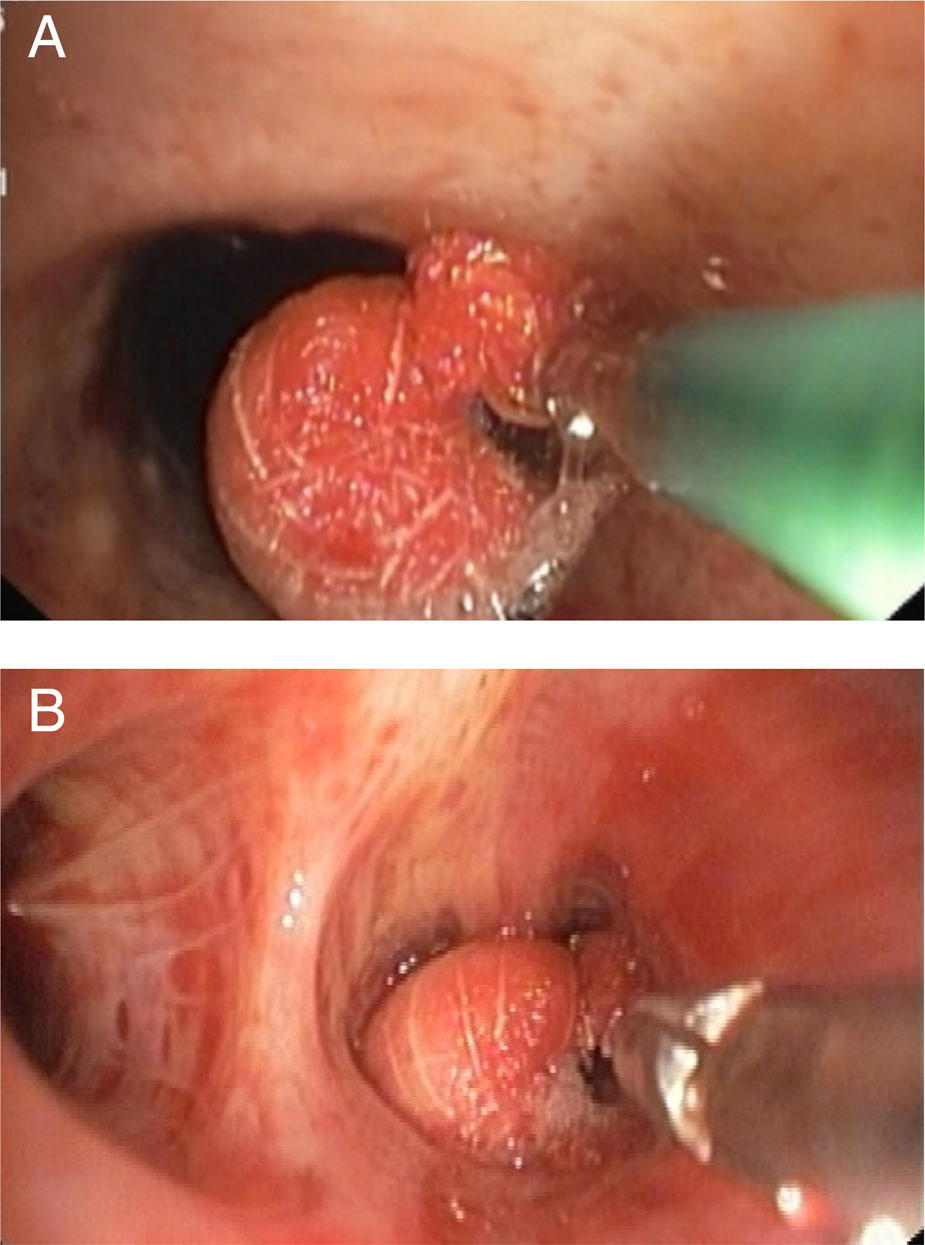
(A) The ​devised spigot being manoeuvred into position with forceps introduced through the working channel of the bronchoscope. (B) The spigot in position, tucked snugly into the bleeding segment.

The in‐hospital course was complicated by worsening haemoptysis threatening to compromise the airway. The patient was intubated with an 8.5‐mm internal diameter endotracheal tube, mechanically ventilated at 40% fraction of inspired oxygen (FiO_2_), and was paralysed with atracurium. The patient was transferred to the medical intensive care unit. Check bronchoscopy performed under conscious sedation showed an active ooze of bleeding along with clots in the left lower lobe posterior segment (Fig. 1B). An emergent therapeutic bronchoscopy was done using the Olympus BF1T 150 (Japan) with an outer diameter of 6 mm and a working channel diameter of 2.8 mm. As there was no access to the conventional EWS and the patient was bleeding incessantly, the team devised an innovative method of using the classic peanut surgical gauze as a suitable alternative. The gauze ball was made up of gauze pads rolled into the shape of a peanut and secured with a regular silk thread tied around its neck (Fig. 2). The gauze tamponade was sprayed with tranexamic acid. A forceps was introduced through the working channel of the bronchoscope with the peanut gauze held at the tip. It was then carefully lowered into the ET tube and manoeuvred into the anterior segment of the left lower lobe so that it would fit snugly in the segment. It resulted in a successful tamponade of the bleeding site (Fig. 3).

Bronchoalveolar lavage (BAL) samples were collected (which showed no bacteria or fungal elements, was negative for organisms/granulomas, and had no growth on culture after 48 h of incubation). The patient was continued on mechanical ventilation under deep sedation and paralytics. Check bronchoscopy performed after two days revealed the in situ peanut gauze. Using forceps, the peanut and clot were cautiously dislodged and removed through the ET tube. No active bleeding was noted and the mucosa at the site of tamponade was healthy. The patient was weaned off the ventilator and extubated to face mask oxygen support. He was observed for the next few days before discharge. The patient had review visits after one week and three and six months, and reported no further episodes of haemoptysis.

## Discussion

### Definition

Life‐threatening haemoptysis (previously known as massive haemoptysis) refers to the expectoration of a large quantity of blood and/or a rapid rate of bleeding. Although there is no consensus on the volume of haemoptysis to assign a grade of severity, 150 mL/24 h or a rate of >100 mL/h is acceptable for practical purposes [4]. In essence, any volume of blood that may lead to airway obstruction or haemodynamic instability is referred to as life‐threatening haemoptysis. Such patients require careful observation in the hospital until the active bleeding has subsided and the underlying cause has been identified and treated.

### Aetiology

The source of bleeding in life‐threatening haemoptysis is the bronchial artery (a high‐pressure circulation) or pulmonary artery (a low‐pressure circulation) in 90% and 5% of the cases, respectively [5]. The remaining 5% of the cases are attributed to non‐bronchial systemic arteries [6]. The most common aetiologies (Table 1) include tuberculosis, bronchiectasis, and bronchogenic carcinoma.

**Table 1:** Aetiology of massive haemoptysis.

Pulmonary
Bronchiectasis
Chronic bronchitis
Pulmonary embolism and infarction
Malignancy
Infectious
Tuberculosis
Fungal infections (aspergillomas and other mycetomas)
Bacterial and viral bronchitis/pneumonia
Necrotizing pneumonia and lung abscess
Parasitic infections
Drug induced
Anticoagulants and antiplatelets
Penicillamine
Bevacizumab
Solvents
Cocaine abuse
Vascular
Vascular malformations (including Dieulafoy's disease)
Pulmonary artery aneurysm
Ruptured thoracic aneurysm
Bronchial artery aneurysm
Pulmonary hypertension
Pulmonary veno‐occlusive disease
Cardiac
Congenital heart disease
Left ventricular failure
Mitral stenosis
Haematological
Platelet disorders
Coagulopathies
Thrombotic thrombocytopenic purpura
Rheumatological
Alveolar haemorrhage due to vasculitis (granulomatosis with polyangiitis, Goodpasture's syndrome, systemic lupus erythematosus, cryoglobulinaemia, rheumatoid arthritis, and Henoch–Schonlein purpura)
Others
Trauma (iatrogenic and blunt/penetrating trauma)
Cryptogenic/idiopathic
Foreign body aspiration
Lung transplantation

However, despite a careful evaluation and use of advanced diagnostic modalities, in up to 40% of the cases (depending on the study), the cause of bleeding cannot be determined. Such cases of cryptogenic haemoptysis have been linked to a high incidence among smokers owing to smoking‐induced inflammatory changes in the bronchial vessels [7]. A French study retrospectively evaluated three‐year outcomes of ~15,000 patients with haemoptysis. Among the study population, 4% of the cases of cryptogenic haemoptysis were diagnosed with lung cancer in the follow‐up period. In a similar case series, 1.5% of the cases of cryptogenic haemoptysis developed lung cancer in the subsequent five years [8, 9].

### Diagnosis

Chest radiography is the initial diagnostic modality of choice owing to its accessibility and availability. It shows the site of bleeding in 45–60% of the cases and the cause of bleeding in 25–30% [1]. Chest radiography, however, is not sensitive and a negative one warrants other diagnostic methods such as CT scan or bronchoscopy. CT scan offers a higher diagnostic yield and is an important tool to guide BAE in massive haemoptysis, whereas bronchoscopy can be performed bedside and used to perform endobronchial procedures.

### Treatment

The management of life‐threatening haemoptysis requires a multidisciplinary approach comprised of pulmonologists, radiologists, and intensivists. The goal of treatment is to arrest the bleeding. Although there are no established guidelines on the same, a flowchart of its management is described by Radchenko et al. [7].

The scope of BAE in our patient was discussed with the interventional radiologist who concluded that there were no discernible culprit vessels on the CT thorax. It precluded the necessity for a bronchial angiogram. BAE is the conventional first‐line therapy for all types of haemoptysis and is generally accepted for evidently abnormal vessels. It has a success rate ranging from 82% to 98% with a long‐term recurrence rate of 10–57% [7]. Studies evaluating its role in cryptogenic haemoptysis have reported successful embolization in 85–95% of the cases. A Korean study described a mild recurrence of haemoptysis in 11.5% of the cryptogenic cases, requiring re‐embolization. However, long‐term follow‐up data on recurrence and the role of BAE in cryptogenic haemoptysis is insufficient [10].

Endobronchial tamponade with silicone spigot was first described by Dutau et al. in 2006. This is an innovative technique that prevents airway flooding and suffocation due to haemoptysis [11]. Initially developed by Watanabe et al. in 1991 [12] for the management of intractable pneumothorax and bronchial fistula, its use has expanded to the treatment of bronchial bleeding. It is usually utilized as a bridge to definitive therapy such as BAE or surgery. One study reported a success rate of 78% with the usage of silicone spigot in patients with moderate haemoptysis [13]. In recent times, its independent role in the radical treatment of idiopathic massive haemoptysis is being considered [14]. It presents with several potential advantages [3]:

- Simple and swift.
- Avoids the risks/complications of BAE.
- In cases of failed BAE or patients with recurrent haemoptysis of unknown origin.
- When BAE facility is unavailable.
- When the patient is haemodynamically unstable and/or has multiple comorbidities.

In the above‐mentioned studies, the spigot was removed after a median of four days to avoid the risk of infection. Most studies on spigot revolve around its use in fistulous lung diseases. Its effectiveness in intractable haemoptysis has to be further explored.

In our patient, the immediate unavailability of the EWS and the presenting circumstance of massive, emergent bleeding prompted an expeditious solution to arrest the same. In our patient, we used a peanut gauze with a silk thread around its neck, an innovative method, which became a suitable and successful alternate for a silicone spigot. It is worth mentioning here that a distinct advantage in our case was that the visibility was extraordinarily good (this is often lacking when there is more endobronchial blood as it may become less clear where the peanut has to be placed). The peanut gauze was left in situ at the site of bleeding as a tamponade for only two days. This forestalled the formation of granulation tissue in the long run. In addition, the gauze tamponade was sprayed with tranexamic acid and functioned as a reservoir preventing the breakdown of any formed clot distal to the occlusion, thus serving a dual purpose in achieving haemostasis. This undoubtedly added an advantage over the usual placement of an EWS. The role of tranexamic acid instilled into the airway via nebulization is a proven technique of arresting massive haemoptysis and has been used in conjunction with other modalities to arrest bleeding [15, 16].

### Conclusion

The placement of a peanut gauze for endobronchial tamponade and treatment of massive haemoptysis is an innovative technique that can be adapted in the future to arrest bleeding localized to a segmental airway. Although the placement of an EWS into the segmental airway to tamponade the bleeding is a fairly straight forward procedure in institutions with expertise and access to flexible bronchoscopy, we were able to further simplify the procedure by replacing the EWS with a peanut gauze. Peanut gauze is not only readily available in any resource‐limited healthcare setup, but also cuts down the relative cost of the procedure for the patient. In addition, this measure serves to temporarily stabilize critically ill patients with massive haemoptysis and acts as a bridge to definitive therapy. This technique requires further experience to gain a deeper understanding of its applicability.

### Disclosure Statement

Appropriate written informed consent was obtained for publication of this case report and accompanying images.

### Author Contribution Statement

Sanjana Chetana Shanmukhappa: Conception, interpretation of data, drafting of work, revision of work, final approval. Srivatsa Lokeshwaran: Conception, interpretation of data, drafting of work, revision of work, final approval. K. Sunil Kumar: Conception, interpretation of data, revision of work, final approval. Prakash Doraiswamy: Conception, interpretation of data, revision of work, final approval.
